# Drive in Sports: How Mental Fatigue Affects Endurance Performance

**DOI:** 10.3389/fpsyg.2018.01383

**Published:** 2018-08-17

**Authors:** Lieke Schiphof-Godart, Bart Roelands, Florentina J. Hettinga

**Affiliations:** ^1^School of Sport, Rehabilitation and Exercise Sciences, University of Essex, Colchester, United Kingdom; ^2^Faculty of Health, Nutrition & Sport, The Hague University of Applied Sciences, The Hague, Netherlands; ^3^Human Physiology Research Group, Vrije Universiteit Brussel, Brussels, Belgium

**Keywords:** motivation, endurance exercise, pacing, cognitive fatigue, perceived exertion

## Abstract

Performance in endurance sports relies on athletes’ drive, which is the sum of all factors pushing athletes to exert effort during exercise. Mental fatigue can influence endurance performance by decreasing athletes’ drive to exercise. From a psychological point of view, mental fatigue has two separate components: it can affect drive by increasing the perceived effort necessary for a given task (“I cannot do this, I am too exhausted”), or by decreasing the perceived value of the reward that can be obtained (“I do not want to do this, it is not worth it”). Neurophysiological theories confirm this dual nature of mental fatigue. It is suggested that mental fatigue can activate the inhibition centers of the brain, increasing perceived effort for a given task, hence decreasing drive and willingness to act. On the other hand, it may also deactivate facilitative brain centers (normally responsible for motivated behavior and increased drive toward a reward), also resulting in decreased drive. In this Perspective we will adopt a multidimensional approach, describing how mental fatigue interacts with drive and performance in endurance exercise. We aim to show how mental fatigue affects endurance performance via two main mechanisms: perceived effort and reward. We will study the interaction between mental fatigue and other factors impacting on drive, such as perceived exertion and motivation, and examine how these factors combined result in athletes’ exercise behavior (such as pacing) and performance. This will provide researchers, coaches, and athletes with useful tools in order to understand, influence and enhance athletes’ drive in exercise, which is of high relevance in elite endurance sports, where mental fatigue, motivation, and stakes all are of the highest level.

## Introduction

Mental fatigue can decrease endurance performance on cognitive and physical tasks (Marcora et al., 2009; Dantzer et al., 2014; MacMahon et al., 2014; Silva-Júnior et al., 2016; McMorris et al., 2018), and impact athletes’ endurance performance by decreasing their drive to exercise, but its mechanism is rarely studied and poorly understood (Martin et al., 2018). Drive is an important determinant of performance in endurance sports, as athletes’ need to push themselves maximally to reach the finish line first, and it is an important factor in athletes’ perseverance during training (Deci and Ryan, 2000; Vallerand, 2012). In this Perspective, we will therefore examine the interaction between mental fatigue, motivation and drive in athletes, as oftentimes either one factor or the other, but rarely all are studied together.

In neurophysiology as well as psychology, the terms ‘motivation’ and ‘drive’ have been used alternately in describing the direction, intensity, and persistence of behavior (Berridge, 2004; Vallerand, 2012). The difference between these constructs is important in discussing factors affecting athletes’ choices and actions during exercise. We propose to use the term motivation to represent each of all different stimuli, acting within or on a person to initiate or discourage behavior. As such, several different motivational factors might act upon an athlete at one given moment in time. We will regard drive as the total of all motivational factors at play, in other words, the resulting ‘drive’ is what pushes an athlete into action. Adopting a multidimensional approach, we will provide a brief overview of the current knowledge regarding the mechanisms through which mental fatigue affects athletes’ drive. A better comprehension of the interaction between relevant factors affecting drive will enable us to understand athletes’ choices and behavior during endurance exercise, may provide tools to influence and enhance their performance and possibly to counteract mental fatigue.

## Mental Fatigue and Subsequent Performance

Mental fatigue manifests primarily as reduced cognitive performance (Chaudhuri and Behan, 2004), and is caused by prolonged periods of demanding cognitive activity (Boksem and Tops, 2008). Mentally fatiguing tasks, such as the ‘Stroop task,’ force subjects to inhibit their initial response before providing the right answer. They are perceived as effortful and fatiguing; and ultimately reduce cognitive efficiency and performance (Chaudhuri and Behan, 2004; Marcora et al., 2009). Task duration seems crucial, as short duration tasks (less than 30 min) have not been found to yield any negative effects on consequent exercise performance, but can impair cognitive performance (Hagger et al., 2010; Graham et al., 2014). The phenomenon of ‘ego-depletion’ is similar to mental fatigue and describes an expected performance decrease after a demanding, mostly inhibition task (e.g., Baumeister et al., 1998). Nevertheless, a recent meta-analysis demonstrated no effect of short duration ‘ego-depleting’ tasks on subsequent endurance exercise performance (Carter and McCullough, 2014).

Mental fatigue is associated with a broad range of ‘side-effects’: lack of energy, increased fatigability and feelings of lassitude, decreased feelings of motivation and alertness and changes in perception and mood (Dantzer et al., 2014). Therefore, it has been suggested that the consequences of mental fatigue may be twofold: it might hamper performance by increasing feelings of fatigue ‘I cannot do it, I am exhausted,’ or by devaluating the importance of success at that specific task: ‘I do not feel like doing it, it is not worth it’ (Dantzer et al., 2014). On the contrary, the ‘strength of self-control theory’ posits that ego-depleting tasks deplete a single global metaphorical strength that has limited capacity and hence impair subsequent performance (Baumeister et al., 1998). Nevertheless, the hypothesis of the depletion of energy substrates (such as glucose) in the brain causing performance decrements has been refuted convincingly (Kurzban, 2010; Friese et al., 2018). Beedie and Lane (2012) suggest convincingly that the effects of mentally fatiguing tasks should be considered in light of an individual’s allocation of resources in response to the perceived importance of the task at hand (and its costs). We therefore propose to study the effects of mental fatigue by considering the interaction between perception of effort and motivation (Kurzban et al., 2013; Pageaux, 2014).

Athletes’ drive is the result of their perception of effort and the perceived value of the reward their activity brings (Dantzer et al., 2014). Mental fatigue might thus affect drive through different pathways. From a neurophysiological perspective, this has been explained by the existence of two separate systems in the brain involved in the regulation of behavior: a mental inhibition and a mental facilitation system (Ishii et al., 2014). The first inhibits athletes’ actions by increasing perceived exertion; the second facilitates their actions by increasing motivation toward a reward (Ishii et al., 2014). Mental fatigue indeed results in increased perceived effort, and has been found to activate the cortical regions involved in athletes’ inhibition system (Marcora et al., 2009; Pageaux, 2014; Van Cutsem et al., 2017a). But mental fatigue may also play a role in deactivating the facilitative system that normally encourages athletes toward action, as it has been found to influence brain regions involved in the cognitive aspect of central motor command as well (Hallett, 2007). From a neurophysiological point of view it is thus plausible that mental fatigue may impact motivation, as it negatively affects readiness to exert physical work in order to obtain a reward (Rudebeck et al., 2006; Walton et al., 2006), although this has not been demonstrated in endurance athletes (Van Cutsem et al., 2017a). Interestingly professional cyclists seem more resistant to mental fatigue than their untrained counterparts (Martin et al., 2016).

### Mental Fatigue and Perception of Effort in Endurance Sports

Mental fatigue causes individuals to perform less well than expected (Dantzer et al., 2014), even in exercise of long duration although cognitive functioning (such as reaction time or complex decision making) seems less crucial for a successful endurance performance (Elferink-Gemser and Hettinga, 2017; Marcora et al., 2009; Van Cutsem et al., 2017a). When mentally fatigued, athletes have been found to perceive endurance exercise as more effortful despite a similar objective power output (Marcora et al., 2009; Pageaux et al., 2013; Pageaux, 2014) and mentally fatigued recreational cyclists showed a faster increase in rate of perceived exertion and impaired performance (Pires et al., 2018). Mentally fatigued athletes consistently choose to perform at lower intensities (compared to a control condition) and to produce less work during self-paced trials (Brownsberger et al., 2013). This decreased drive explains the deleterious effect of mental fatigue on endurance performance (Marcora and Staiano, 2010; Roelands et al., 2013; Pageaux, 2016): mental fatigue increases the feeling ‘I cannot do it, I am exhausted’ (Dantzer et al., 2014), corresponding to an activation of the inhibition system in the brain, as proposed by Ishii and colleagues (Ishii et al., 2014).

#### Perception of Effort From a Biopsychological Perspective

After mentally fatiguing tasks, modifications in electric brain activity patterns (EEG) have been observed (Käthner et al., 2014; Wascher et al., 2014), and these altered brain patterns are related to increased perceived exertion (Brownsberger et al., 2013). Alterations in brain activation and the concurrent changes in brain neurotransmitter concentrations mediate between athletes’ perceptions (for example of mental fatigue) and their drive to exercise (Meeusen et al., 2006; Roelands et al., 2013). Prolonged neural activity inducing mental fatigue can increase brain adenosine concentrations (Lovatt et al., 2012), which in turn decrease drive (Davis et al., 2003; Martin et al., 2018). Serotonin, another neurotransmitter, is related to increased sensitivity to negative stimuli (such as perceived fatigue or effort), and also increases perceived exertion (Roelands and Meeusen, 2010; Hebart and Gläscher, 2015). Additionally, neurotransmitters such as dopamine and serotonin are involved in feelings of motivation or the lack thereof (amotivation) (Meeusen et al., 2006; Roelands et al., 2008). Martin et al. (2018) recently suggested that the accumulation of extracellular cerebral adenosine may serve to explain the relationship between mental fatigue and exercise performance by increasing perception of effort during subsequent effortful tasks, and by impairing motivation (Martin et al., 2018). While there is clear evidence that mental fatigue impacts perceived exertion (Marcora et al., 2009), it seems also relevant to examine whether and how mental fatigue influences athletes’ motivation toward exercise and which factors may interfere in this relationship.

### Mental Fatigue and Perception of Reward in Endurance Sports

It has often been suggested that mental fatigue impacts endurance performance by decreasing motivation (Chaudhuri and Behan, 2004; Marcora and Staiano, 2010; Pageaux et al., 2013; Dantzer et al., 2014; Van Cutsem et al., 2017a; Martin et al., 2018). Motivation toward short and long term goals is of great importance concerning drive and performance in endurance sports, and has been studied extensively from a psychological point of view (McCormick et al., 2015; Clancy et al., 2016). Elite athletes consistently report higher motivation and commitment than non-elites (Halldorsson et al., 2012), while a lack of motivation (amotivation) is related to worse performance (Mouratidis et al., 2008) and extreme motivation, for example in the form of obsessive passion, leads to excessive drive to exercise (Mageau et al., 2009; Vallerand, 2012; Schiphof-Godart and Hettinga, 2017).

According to sports psychology theories, motivation and drive depend on athletes’ choice of goals and their persistence toward those (Nicholls, 1984; Wigfield and Eccles, 2000; Braver et al., 2014; Dweck, 2017). Different types of goals and perceived rewards (e.g., outperforming oneself or winning a race), explain different types of motivation. A distinction can be made between extrinsic motivation (toward, for example, monetary rewards) and intrinsic motivation for the pleasure an activity itself represents (Deci and Ryan, 2000; Dweck, 2017), and between primary (long term) goals versus secondary, short term objectives. Athletes may demonstrate a particularly strong persistence in activities that fulfill lifelong goals, such as the protection of self-esteem and the fulfillment of basic psychological needs (Deci and Ryan, 2000; Dweck, 2017). These needs to feel autonomous, competent and related to significant others, can be seen as the ultimate origin of athletes’ drive (Deci and Ryan, 2000). Until now, no effect of mental fatigue on reported intrinsic motivation or desire to succeed in an exercise task has been reported in athletes (Martin et al., 2018; Van Cutsem et al., 2017a), but most studies have focussed mainly on mental fatigue and perceived exertion, and might not have been able to detect subtle changes in the magnitude or nature of athletes’ motivation (Van Cutsem et al., 2017a). A neurophysiological approach might thus be valuable in providing plausible mechanisms, if any, through which mental fatigue might affect athletes’ motivation.

#### Perception of Rewards From a Biopsychological Perspective

The neurotransmitter dopamine can activate brain regions that make a reward seem more valuable (Volkow et al., 2004; Meeusen et al., 2006; Roelands et al., 2008; Braver et al., 2014), and thus influence athletes’ drive to exercise (Boksem et al., 2006; Meeusen et al., 2007; Pageaux, 2014; Zając et al., 2015; Barte et al., 2017). Elevated dopamine concentrations in the brain can ‘push’ athletes toward action and exerting effort (Roelands et al., 2008) and influence the decision ‘not to give up,’ especially under difficult circumstances (Roelands et al., 2015). Dopamine increases athletes’ attention toward rewarding goals, and strengthens their reaction to the positive feelings these can provide (Roelands et al., 2008). Indications exist that mental fatigue inversely affects the same ‘facilitative’ brain pathways as does dopamine (Ishii et al., 2014; Martin et al., 2018), and thus has an opposite effect. While mental fatigue increases adenosine concentration in the brain (which decreases dopamine levels), caffeine and other substances can inverse the neurotransmitter balance and thus counteract the effects of mental fatigue (Dunwiddie and Masino, 2001; Lorist and Tops, 2003; Azevedo et al., 2016).

## Mental Fatigue and Pacing in Endurance Sports

Pacing is the term used in endurance sports to represent an athlete’s choice regarding the amount of effort exerted (Abbiss and Laursen, 2008; Roelands et al., 2013; Pageaux, 2014). The best pacing strategy would imply an optimal use of energy resources during exercise, and results in the best possible performance (Foster 2003 of 2004, Skorski and Abbiss, 2017). Performance in endurance sports therefore does not only reflect physical skills, but also relies on decision making during a race (Smits et al., 2014; Micklewright et al., 2017). Pacing can be altered in various ways: by mentally fatiguing tasks before a race, presenting athletes with an opponent, (Viru et al., 2010; Hettinga et al., 2017; Konings et al., 2017; Konings and Hettinga, 2017), music (Terry et al., 2012; Laukka and Quick, 2013; Elvers and Steffens, 2017), by using self-talk (Hatzigeorgiadis et al., 2011; Blanchfield et al., 2014), or providing feedback or (monetary) rewards (Hulleman et al., 2007; Skorski and Abbiss, 2017).

### Pacing and Performance in Endurance Sports and the Perception of Effort Versus Rewards

Both the mental inhibition and the facilitative system are involved in athletes’ drive and exercise behavior (Dantzer et al., 2014; Ishii et al., 2014). Interventions lowering perception of effort (by decreasing inhibition), for example by manipulation of brain neurotransmitters, improve performance (Roelands et al., 2013), and, contrarily, increases in perceived exertion decrease performance in endurance sports by causing an altered, suboptimal, pacing strategy (Hampson et al., 2001; Paterson and Marino, 2004; Roelands et al., 2013). On the other hand, increases in intrinsic motivation also increase athletes’ drive and consequently enhance pacing strategies and performance, probably not affecting athletes’ inhibition system, but acting on the brain’s facilitation system instead (Deci and Ryan, 2000).

Several environmental factors, such as opponents or supporters, can influence perception of effort or perception of rewards (motivation), and consequently enhance pacing and performance through one or both brain systems (Cerasoli et al., 2014; Smits et al., 2014; Abbiss et al., 2015; Hettinga et al., 2017; Konings et al., 2017). Athletes’ intrinsic motivation can be stimulated by increasing their pleasure in an activity, for example by means of music, encouragements (Nakamura et al., 2010; Laukka and Quick, 2013; Loizou and Karageorghis, 2014), positive feedback (Harackiewicz, 1979; Mouratidis et al., 2008), fun and a challenge such as racing against an opponent (de Franco Tobar et al., 2013; Konings et al., 2017). Extrinsic motivation (for example by external rewards such as money), sometimes increases, but also often decreases performance (Harackiewicz, 1979; Lander et al., 2009; Hagger and Chatzisarantis, 2011; Cerasoli et al., 2014; Barte et al., 2017), and athletes may even experience both types of motivation at the same time. The prospective of winning a golden medal may represent both an extrinsic (money, fame…) and intrinsic (enhanced feelings of competence) motivation (Deci and Ryan, 2000; Dweck, 2017), and some interventions may affect both perceived exertion and perceived reward. Providing athletes with an opponent, for example, may enable them to race faster while maintaining the same perception of effort, even when they are physically fatigued (Konings et al., 2017), and a higher reward or cup of coffee may counteract at least partly the effects of mental fatigue on perceived exertion (Barte et al., 2017; Van Cutsem et al., 2017b). Hence, factors affecting either perception of effort or perception of reward may interact (St Clair Gibson et al., 2017), and the result may prove unpredictable until now.

## Mental Fatigue and Endurance Performance in Sports

### Endurance Exercise as the Ultimate ‘Field Lab’

Endurance sports provide the ultimate arena for gaining a deeper understanding of how and when mental fatigue influences performance. Only in endurance exercise, the interaction between mental fatigue and motivation can be studied: mental fatigue seems not to affect athletes’ maximal strength, explosive power, and anaerobic work (Boksem et al., 2006; Dantzer et al., 2014; Martin et al., 2015; Van Cutsem et al., 2017a).

The reason for this may lie in the different mechanisms causing fatigue and obliging athletes to adapt their power output in tasks of long and short duration (Gandevia, 2001). Short duration, anaerobic exercise is mostly limited by peripheral fatigue (such as the availability of energy-providing substrates) (Coggan and Coyle, 1991), but in endurance exercise, a reduction in central motor drive and central fatigue are observed (Amann, 2011). Thus, in endurance exercise, the brain decides when to quit, in short duration exercise the muscles do (Gandevia, 2001). Mental fatigue may disturb the decision making-process involved in pacing, affecting the cognitive control necessary to choose an optimal pacing strategy, while an all-out strategy does not involve such ‘ thinking’ (Martin et al., 2018): the brain does not need much cognitive effort for a one-second task requiring maximal strength. Although highly motivated athletes may find ways to compensate for the effects of mental fatigue, these compensation strategies may not be sustainable on the long term (Boksem et al., 2006). Maintaining performance when mentally fatigued necessitates more effort (‘thinking harder’), or adaptive strategies: often precision on a task needs to be sacrificed for speed (or vice versa), and these strategies might not always be available during long duration exercise (Boksem et al., 2006).

Mental fatigue can be counteracted: Rinsing the mouth with a carbohydrate (maltodextrin) and caffeine drink (without swallowing it) during a long duration Stroop task, reduces feelings of mental fatigue and its effects on a cognitive test (Van Cutsem et al., 2017b). Increased dopamine concentrations seem to negate the effects of mental fatigue by decreasing brain adenosine concentrations (Azevedo et al., 2016; Van Cutsem et al., 2017b). High dopamine levels allow athletes to produce more work and endure higher body temperatures compared to unaltered dopamine levels, while the athletes rate their effort and feelings of warmth as lower (Watson et al., 2005; Roelands et al., 2008; Cordery et al., 2017), and an added monetary reward or the taste of a good cup of coffee might do the same (Hulleman et al., 2007; Barte et al., 2017; Skorski and Abbiss, 2017; Van Cutsem et al., 2017b).

### Practical Advice for Researchers and Coaches

Athletes’ drive can thus be increased, and mental fatigue can be decreased, by manipulating athletes’ intrinsic motivation, the value of a reward or directly by altering brain neurotransmitter concentrations. How these strategies affect athletes’ drive to exercise nevertheless remains largely unknown. Strategies affecting drive to exercise, for example providing athletes with feedback or an opponent, may work through a diminished cognitive load (athletes do not need to think about an optimal ‘pace’), an increased or decreased perceived load (caused by a forced rather than self-chosen pace or thanks to distraction) or increased intrinsic motivation (for example by increasing feelings of competence) (Lander et al., 2009; Brick et al., 2016). Nevertheless, how exactly these strategies affect athletes’ drive to exercise and their choice to allocate the resources needed to continue or even increase their effort remains largely unknown (Beedie and Lane, 2012; Kurzban et al., 2013). More multidisciplinary research thus seems useful to disentangle the effects of ‘motivating’ manipulations in endurance exercise, while taking mental fatigue and its effect on the balance between effort and rewards, into account.

### Concluding Remarks

Future studies of mental fatigue and its impact on athletes’ drive might benefit from considering a psychobiological approach. The effect of mental fatigue should be examined regarding athletes’ perceived effort (and the activation of inhibition centers in the brain), in combination with modifications in their motivation toward a reward (and the deactivation of facilitative brain centers). A better understanding of both components of mental fatigue will be of benefit in both research and practice, as it will enable the manipulation and optimalisation of endurance performance. Elite endurance sports represent an excellent arena to do just that, as mental strain and stress are elevated, stakes are high, and athletes might already possess sky-high motivation toward obtaining their Olympic title.

## Author Contributions

LS-G wrote the article. LS-G and FH conceptualized and revised the paper critically for important intellectual content, final approval of the version to be published, and accountability for all aspects of the work. BR revised the paper critically for important intellectual content, final approval of the version to be published, and accountability for all aspects of the work.

## Conflict of Interest Statement

The authors declare that the research was conducted in the absence of any commercial or financial relationships that could be construed as a potential conflict of interest. The handling Editor declared a shared affiliation, though no other collaboration, with one of the authors LS-G at the time of review.

## References

[B1] Abbiss C. R. Laursen P. B. (2008). Describing and understanding pacing strategies during athletic competition. *Sports Med.* 38 239–252. 10.2165/00007256-200838030-00004 18278984

[B2] Abbiss C. R. Peiffer J. J. Meeusen R. Skorski S. (2015). Role of ratings of perceived exertion during self-paced exercise: what are we actually measuring? *Sports Med.* 45 1235–1243. 10.1007/s40279-015-0344-5 26054383

[B3] Amann M. (2011). Central and peripheral fatigue: interaction during cycling exercise in humans. *Med. Sci. Sports Exerc.* 43 2039–2045. 10.1249/MSS.0b013e31821f59ab 21502884

[B4] Azevedo R. Silva-Cavalcante M. D. Gualano B. Lima-Silva A. E. Bertuzzi R. (2016). Effects of caffeine ingestion on endurance performance in mentally fatigued individuals. *Eur. J. Appl. Physiol.* 116 2293–2303. 10.1007/s00421-016-3483-y 27695980

[B5] Barte J. C. M. Nieuwenhuys A. Geurts S. A. E. Kompier M. A. J. (2017). Fatigue experiences in competitive soccer: development during matches and the impact of general performance capacity. *Fatigue* 5 191–201. 10.1080/21641846.2017.1377811

[B6] Baumeister R. F. Bratslavsky E. Muraven M. Tice D. M. (1998). Egodepletion: is the active self a limited resource? *J. Pers. Soc. Psychol.* 74 1252–1265. 10.1037/0022-3514.74.5.1252 9599441

[B7] Beedie C. J. Lane A. M. (2012). The role of glucose in self-control: Another look at the evidence and an alternative conceptualization. *Pers. Soc. Psychol. Rev.* 16 143–153. 10.1177/1088868311419817 21896791

[B8] Berridge K. C. (2004). Motivation concepts in behavioral neuroscience. *Physiol. Behav.* 81 179–209. 10.1016/j.physbeh.2004.02.004 15159167

[B9] Blanchfield A. W. Hardy J. De Morree H. M. Staiano W. Marcora S. M. (2014). Talking yourself out of exhaustion: the effects of self-talk on endurance performance. *Med. Sci. Sports Exerc.* 46 998–1007. 10.1249/MSS.0000000000000184 24121242

[B10] Boksem M. A. Meijman T. F. Lorist M. M. (2006). Mental fatigue, motivation and action monitoring. *Biol. Psychol.* 72 123–132. 10.1016/j.biopsycho.2005.08.007 16288951

[B11] Boksem M. A. Tops M. (2008). Mental fatigue: costs and benefits. *Brain Res. Rev.* 59 125–139. 10.1016/j.brainresrev.2008.07.001 18652844

[B12] Braver T. S. Krug M. K. Chiew K. S. Kool W. Westbrook J. A. Clement N. J. (2014). Mechanisms of motivation–cognition interaction: challenges and opportunities. *Cogn. Affect. Behav. Neurosci.* 14 443–472. 10.3758/s13415-014-0300-0 24920442 PMC4986920

[B13] Brick N. E. Campbell M. J. Metcalfe R. S. Mair J. L. Macintyre T. E. (2016). Altering pace control and pace regulation: attentional focus effects during running. *Med. Sci. Sports Exerc.* 2016 879–886. 10.1249/MSS.0000000000000843 26673128

[B14] Brownsberger J. Edwards A. Crowther R. Cottrell D. (2013). Impact of mental fatigue on self-paced exercise. *Int. J. Sports Med.* 34 1029–1036. 10.1055/s-0033-1343402 23771830

[B15] Carter E. C. McCullough M. E. (2014). Publication bias and the limited strength model of self-control: has the evidence for ego depletion been overestimated? *Front. Psychol.* 5:823. 10.3389/fpsyg.2014.00823 25126083 PMC4115664

[B16] Cerasoli C. P. Nicklin J. M. Ford M. T. (2014). Intrinsic motivation and extrinsic incentives jointly predict performance: a 40-year meta-analysis. *Psychol. Bull.* 140 980–1008. 10.1037/a0035661 24491020

[B17] Chaudhuri A. Behan P. O. (2004). Fatigue in neurological disorders. *Lancet* 363 978–988. 10.1016/S0140-6736(04)15794-2 15043967

[B18] Clancy R. B. Herring M. P. MacIntyre T. E. Campbell M. J. (2016). A review of competitive sport motivation research. *Psychol. Sport Exerc.* 27 232–242. 10.1016/j.psychsport.2016.09.003

[B19] Coggan A. R. Coyle E. F. (1991). Carbohydrate ingestion during prolonged exercise: effects on metabolism and performance. *Exerc. Sports Sci. Rev.* 19 1–40. 10.1249/00003677-199101000-00001 1936083

[B20] Cordery P. Peirce N. Maughan R. J. Watson P. (2017). Dopamine/noradrenaline reuptake inhibition in women improves endurance exercise performance in the heat. *Scand. J. Med. Sci. Sports* 27 1221–1230. 10.1111/sms.12753 27739188

[B21] Dantzer R. Heijnen C. J. Kavelaars A. Laye S. Capuron L. (2014). The neuroimmune basis of fatigue. *Trends Neurosci.* 37 39–46. 10.1016/j.tins.2013.10.003 24239063 PMC3889707

[B22] Davis J. M. Zhao Z. Stock H. S. Mehl K. A. Buggy J. Hand G. A. (2003). Central nervous system effects of caffeine and adenosine on fatigue. *Am. J. Physiol. Regul. Integr. Comp. Physiol. Rev.* 284 R399–R404. 10.1152/ajpregu.00386.2002 12399249

[B23] de Franco Tobar L. Meurer S. T. Benedetti T. B. (2013). Motivational factors of senior athletes to participate in the Ironman. *Sci. Sports* 28 e63–e65. 10.1016/j.scispo.2013.01.001

[B24] Deci E. L. Ryan R. M. (2000). The” what” and” why” of goal pursuits: human needs the self-determination of behavior. *Psychol. Inq.* 11 227–268. 10.1080/08870440902783628 20204932

[B25] Dunwiddie T. V. Masino S. A. (2001). The role and regulation of adenosine in the central nervous system. *Annu. Rev. Neurosci.* 24 31–55. 10.1146/annurev.neuro.24.1.31 11283304

[B26] Dweck C. S. (2017). From needs to goals and representations: foundations for a unified theory of motivation, personality, and development. *Psychol. Rev.* 124 689–719. 10.1037/rev0000082 28933872

[B27] Elferink-Gemser M. T. Hettinga F. J. (2017). Pacing and self-regulation: important skills for talent development in endurance sports. *Int. J. Sports Physiol. Perform.* 12 831–835. 10.1123/ijspp.2017-0080 28605209

[B28] Elvers P. Steffens J. (2017). The sound of success: investigating cognitive and behavioral effects of motivational music in sports. *Front. Psychol.* 8:2026. 10.3389/fpsyg.2017.02026 29209257 PMC5702473

[B29] Friese M. Loschelder D. Gieseler K. Frankenbach J. Inzlicht M. (2018). Is ego depletion real? An analysis of arguments. *Pers. Soc. Psychol. Rev.* 10.1177/1088868318762183 [Epub ahead of print]. 29591537

[B30] Gandevia S. C. (2001). Spinal and supraspinal factors in human muscle fatigue. *Physiol. Rev.* 81 1725–1789. 10.1152/physrev.2001.81.4.1725 11581501

[B31] Graham J. D. Sonne M. W. Bray S. R. (2014). It wears me out just imagining it! Mental imagery leads to muscle fatigue and diminished performance of isometric exercise. *Biol. Psychol.* 103 1–6. 10.1016/j.biopsycho.2014.07.018 25093627

[B32] Hagger M. S. Chatzisarantis N. L. (2011). Causality orientations moderate the undermining effect of rewards on intrinsic motivation. *J. Exp. Soc. Psychol.* 47 485–489. 10.1016/j.jesp.2010.10.010

[B33] Hagger M. S. Wood C. Stiff C. Chatzisarantis N. L. (2010). Ego depletion and the strength model of self-control: a meta-analysis. *Psychol. Bull.* 136 495–525. 10.1037/a0019486 20565167

[B34] Halldorsson V. Helgason A. Thorlindsson T. (2012). Attitudes, commitment and motivation amongst Icelandic elite athletes. *Int. J. Sport Psychol.* 43 241–254.

[B35] Hallett M. (2007). Volitional control of movement: the physiology of free will. *Clin. Neurophysiol.* 118 1179–1192. 10.1016/j.clinph.2007.03.019 17466580 PMC1950571

[B36] Hampson D. B. Gibson A. S. C. Lambert M. I. Noakes T. D. (2001). The influence of sensory cues on the perception of exertion during exercise and central regulation of exercise performance. *Sports Med.* 31 935–952. 10.2165/00007256-200131130-00004 11708402

[B37] Harackiewicz J. M. (1979). The effects of reward contingency and performance feedback on intrinsic motivation. *J. Pers. Soc. Psychol.* 37 1352–1363. 10.1080/00221309.1991.9711135 28147904

[B38] Hatzigeorgiadis A. Zourbanos N. Galanis E. Theodorakis Y. (2011). Self-talk and sports performance: a meta-analysis. *Perspect. Psychol. Sci.* 6 348–356. 10.1177/1745691611413136 26167788

[B39] Hebart M. N. Gläscher J. (2015). Serotonin and dopamine differentially affect appetitive and aversive general Pavlovian-to-instrumental transfer. *Psychopharmacology* 232 437–451. 10.1007/s00213-014-3682-3 25034118

[B40] Hettinga F. J. Konings M. J. Pepping G. J. (2017). The science of racing against opponents: affordance competition and the regulation of exercise intensity in head-to-head competition. *Front. Physiol.* 8:118. 10.3389/fphys.2017.00118 28293199 PMC5329012

[B41] Hulleman M. De Koning J. J. Hettinga F. J. Foster C. (2007). The effect of extrinsic motivation on cycle time trial performance. *Med. Sci. Sports Exerc.* 39 709–715. 10.1249/mss.0b013e31802eff36 17414810

[B42] Ishii A. Tanaka M. Watanabe Y. (2014). Neural mechanisms of mental fatigue. *Rev. Neurosci.* 25 469–479. 10.1515/revneuro-2014-0028 24926625

[B43] Käthner I. Wriessnegger S. C. Müller-Putz G. R. Kübler A. Halder S. (2014). Effects of mental workload and fatigue on the P300, alpha and theta band power during operation of an ERP (P300) brain–computer interface. *Biol. Psychol.* 102 118–129. 10.1016/j.biopsycho.2014.07.014 25088378

[B44] Konings M. J. Hettinga F. J. (2017). Objectifying tactics: athlete and race variability in elite short-track speed skating. *Int. J. Sports Physiol. Perform.* 13 170–175. 10.1123/ijspp.2016-0779 28530528

[B45] Konings M. J. Parkinson J. Zijdewind I. Hettinga F. J. (2017). Racing an opponent alters pacing, performance and muscle force decline, but not RPE. *Int. J. Sports Physiol. Perform.* 13 283–289. 10.1123/ijspp.2017-0220 28657853

[B46] Kurzban R. (2010). Does the brain consume additional glucose during self-control tasks? *Evol. Psychol.* 8 244–259. 22947794 10.1177/147470491000800208 PMC10480967

[B47] Kurzban R. Duckworth A. Kable J. W. Myers J. (2013). An opportunity cost model of subjective effort and task performance. *Behav. Brain Sci.* 36 661–679. 10.1017/S0140525X12003196 24304775 PMC3856320

[B48] Lander P. J. Butterly R. J. Edwards A. M. (2009). Self-paced exercise is less physically challenging than enforced constant pace exercise of the same intensity: influence of complex central metabolic control. *Br. J. Sports Med.* 43 789–795. 10.1136/bjsm.2008.056085 19196729

[B49] Laukka P. Quick L. (2013). Emotional and motivational uses of music in sports and exercise: a questionnaire study among athletes. *Psychol. Music* 41 198–215. 10.1177/0305735611422507

[B50] Loizou G. Karageorghis C. I. (2014). Effects of psychological priming, video, and music on anaerobic exercise performance. *Scand. J. Med. Sci. Sports* 25 909–920. 10.1111/sms.12391 25556962

[B51] Lorist M. M. Tops M. (2003). Caffeine, fatigue, and cognition. *Brain Cogn.* 53 82–94. 10.1016/S0278-2626(03)00206-9 14572506

[B52] Lovatt D. Xu Q. Liu W. Takano T. Smith N. A. Schnermann J. (2012). Neuronal adenosine release, and not astrocytic AtP release, mediates feedback inhibition of excitatory activity. *Proc. Natl. Acad. Sci. U.S.A.* 109 6265–6270. 10.1073/pnas.1120997109 22421436 PMC3341061

[B53] MacMahon C. Schücker L. Hagemann N. Strauss B. (2014). Cognitive fatigue effects on physical performance during running. *J. Sport Exerc. Psychol.* 36 375–381. 10.1123/jsep.2013-0249 25226606

[B54] Mageau G. A. Vallerand R. J. Charest J. Salvy S. J. Lacaille N. Bouffard T. (2009). On the development of harmonious and obsessive passion: the role of autonomy support, activity specialization, and identification with the activity. *J. Pers.* 77 601–646. 10.1111/j.1467-6494.2009.00559.x 20078732

[B55] Marcora S. M. Staiano W. (2010). The limit to exercise tolerance in humans: mind over muscle? *Eur. J. Appl. Physiol.* 109 763–770. 10.1007/s00421-010-1418-6 20221773

[B56] Marcora S. M. Staiano W. Manning V. (2009). Mental fatigue impairs physical performance in humans. *J. Appl. Physiol.* 106 857–864. 10.1152/japplphysiol.91324.2008 19131473

[B57] Martin K. Meeusen R. Thompson K. G. Keegan R. Rattray B. (2018). Mental fatigue impairs endurance performance: a physiological explanation. *Sports Med.* 48 2041–2051. 10.1007/s40279-018-0946-9 29923147

[B58] Martin K. Staiano W. Menaspà P. Hennessey T. Marcora S. Keegan R. (2016). Superior inhibitory control and resistance to mental fatigue in professional road cyclists. *PLoS One* 11:e0159907. 10.1371/journal.pone.0159907 27441380 PMC4956323

[B59] Martin K. Thompson K. G. Keegan R. Ball N. Rattray B. (2015). Mental fatigue does not affect maximal anaerobic exercise performance. *Eur. J. Appl. Physiol.* 115 715–725. 10.1007/s00421-014-3052-1 25425259

[B60] McCormick A. Meijen C. Marcora S. (2015). Psychological determinants of whole-body endurance performance. *Sports Med.* 45 997–1015. 10.1007/s40279-015-0319-6 25771784 PMC4473096

[B61] McMorris T. Barwood M. Hale B. J. Dicks M. Corbett J. (2018). Cognitive fatigue effects on physical performance: a systematic review and meta-analysis. *Physiol. Behav.* 188 103–107. 10.1016/j.physbeh.2018.01.029 29408319

[B62] Meeusen R. Watson P. Hasegawa H. Piacentini M. F. (2006). Central fatigue. The serotonin hypothesis and beyond. *Sports Med.* 36 881–909. 10.2165/00007256-200636100-00006 17004850

[B63] Meeusen R. Watson P. Hasegawa H. Roelands B. Piacentini M. F. (2007). Brain neurotransmitters in fatigue and overtraining. *Appl. Physiol. Nutr. Metab.* 32 857–864. 10.1139/H07-080 18059610

[B64] Micklewright D. Kegerreis S. Raglin J. Hettinga F. (2017). Will the conscious–subconscious pacing quagmire help elucidate the mechanisms of self-paced exercise? New opportunities in dual process theory and process tracing methods. *Sports Med.* 47 1231–1239. 10.1007/s40279-016-0642-6 27778303

[B65] Mouratidis A. Vansteenkiste M. Lens W. Sideridis G. (2008). The motivating role of positive feedback in sport and physical education: evidence for a motivational model. *J. Sport Exerc. Psychol.* 30 240–268. 10.1123/jsep.30.2.240 18490793

[B66] Nakamura P. M. Pereira G. Papini C. B. Nakamura F. Y. Kokubun E. (2010). Effects of preferred and nonpreferred music on continuous cycling exercise performance. *Percept. Mot. Skills* 110 257–264. 10.2466/pms.110.1.257-264 20391890

[B67] Nicholls J. G. (1984). Achievement motivation: conceptions of ability, subjective experience, task choice, and performance. *Psychol. Rev.* 91 328–346. 10.1037/0033-295X.91.3.328

[B68] Pageaux B. (2014). The psychobiological model of endurance performance: an effort-based decision-making theory to explain self-paced endurance performance. *Sports Med.* 44 1319–1320. 10.1007/s40279-014-0198-2 24809249

[B69] Pageaux B. (2016). Perception of effort in exercise science: definition, measurement and perspectives. *Eur. J. Sport Sci.* 16 885–894. 10.1080/17461391.2016.1188992 27240002

[B70] Pageaux B. Marcora S. M. Lepers R. (2013). Prolonged mental exertion does not alter neuromuscular function of the knee extensors. *Med. Sci. Sports Exerc.* 45 2254–2264. 10.1249/MSS.0b013e31829b504a 23698244

[B71] Paterson S. Marino F. E. (2004). Effect of deception of distance on prolonged cycling performance. *Percept. Mot. Skills* 98 1017–1026. 10.2466/pms.98.3.1017-1026 15209319

[B72] Pires F. O. Silva-Júnior F. L. Brietzke C. Franco-Alvarenga P. E. Pinheiro F. A. de França N. M. (2018). Mental fatigue alters cortical activation and psychological responses, impairing performance in a distance-based cycling trial. *Front. Physiol.* 9:227. 10.3389/fphys.2018.00227 29615923 PMC5864900

[B73] Roelands B. de Koning J. Foster C. Hettinga F. Meeusen R. (2013). Neurophysiological determinants of theoretical concepts and mechanisms involved in pacing. *Sports Med.* 43 301–311. 10.1007/s40279-013-0030-4 23456493

[B74] Roelands B. De Pauw K. Meeusen R. (2015). Neurophysiological effects of exercise in the heat. *Scand. J. Med. Sci. Sports* 25(Suppl. 1) 65–78. 10.1111/sms.12350 25943657

[B75] Roelands B. Hasegawa H. Watson P. Piacentini M. F. Buyse L. De Schutter G. (2008). The effects of acute dopamine reuptake inhibition on performance. *Med. Sci. Sports Exerc.* 40 879–885. 10.1249/MSS.0b013e3181659c4d 18408610

[B76] Roelands B. Meeusen R. (2010). Alterations in central fatigue by pharmacological manipulations of neurotransmitters in normal and high ambient temperature. *Sports Med.* 40 229–246. 10.2165/11533670-000000000-00000 20199121

[B77] Rudebeck P. H. Walton M. E. Smyth A. N. Bannerman D. M. Rushworth M. F. (2006). Separate neural pathways process different decision costs. *Nat. Neurosci.* 9 1161–1168. 10.1038/nn1756 16921368

[B78] Schiphof-Godart L. Hettinga F. J. (2017). Passion and pacing in endurance performance. *Front. Physiol.* 8:83. 10.3389/fphys.2017.00083 28265245 PMC5317098

[B79] Silva-Júnior F. L. Emanuel P. Sousa J. Silva M. Teixeira S. Pires F. O. (2016). Prior acute mental exertion in exercise and sport. *Clin. Pract. Epidemiol. Ment. Health* 12 94–107. 10.2174/1745017901612010094 27867415 PMC5095890

[B80] Skorski S. Abbiss C. R. (2017). The manipulation of pace within endurance sport. *Front. Physiol.* 8:102 10.3389/fphys.2017.00102 PMC5326767 28289392

[B81] Smits B. L. Pepping G. J. Hettinga F. J. (2014). Pacing and decision making in sport and exercise: the roles of perception and action in the regulation of exercise intensity. *Sports Med.* 44 763–775. 10.1007/s40279-014-0163-0 24706362

[B82] St Clair Gibson A. Swart J. Tucker R. (2017). The interaction of psychological and physiological homeostatic drives and role of general control principles in the regulation of physiological systems, exercise and the fatigue process–The Integrative Governor theory. *Eur. J. Sport Sci.* 8 25–36. 28478704 10.1080/17461391.2017.1321688

[B83] Terry P. C. Karageorghis C. I. Saha A. M. D’Auria S. (2012). Effects of synchronous music on treadmill running among elite triathletes. *J. Sci. Med. Sport* 15 52–57. 10.1016/j.jsams.2011.06.003 21803652

[B84] Vallerand R. J. (2012). From motivation to passion: in search of the motivational processes involved in a meaningful life. *Can. Psychol.* 53 42–52. 10.1037/a0026377

[B85] Van Cutsem J. De Pauw K. Marcora S. Meeusen R. Roelands B. (2017a). A caffeine-maltodextrin mouth rinse counters mental fatigue. *Psychopharmacology* 235 947–958. 10.1007/s00213-017-4809-0 29247343

[B86] Van Cutsem J. Marcora S. De Pauw K. Bailey S. Meeusen R. Roelands B. (2017b). The effects of mental fatigue on physical performance: a systematic review. *Sports Med.* 47 1569–1588. 10.1007/s40279-016-0672-0 28044281

[B87] Viru M. Hackney A. Karelson K. Janson T. Kuus M. Viru A. (2010). Competition effects on physiological responses to exercise: performance, cardiorespiratory and hormonal factors. *Acta Physiol. Hung.* 97 22–30. 10.1556/APhysiol.97.2010.1.3 20233687

[B88] Volkow N. D. Wang G. J. Fowler J. S. Telang F. Maynard L. Logan J. (2004). Evidence that methylphenidate enhances the saliency of a mathematical task by increasing dopamine in the human brain. *Am. J. Psychiatry* 161 1173–1180. 10.1176/appi.ajp.161.7.1173 15229048

[B89] Walton M. E. Kennerley S. W. Bannerman D. M. Phillips P. E. Rushworth M. F. (2006). Weighing up the benefits of work: behavioral and neural analyses of effort-related decision making. *Neural Netw.* 19 1302–1314. 10.1016/j.neunet.2006.03.005 16949252 PMC2519033

[B90] Wascher E. Rasch B. Sänger J. Hoffmann S. Schneider D. Rinkenauer G. (2014). Frontal theta activity reflects distinct aspects of mental fatigue. *Biol. Psychol.* 96 57–65. 10.1016/j.biopsycho.2013.11.010 24309160

[B91] Watson P. Hasegawa H. Roelands B. Piacentini M. F. Looverie R. Meeusen R. (2005). Acute dopamine/noradrenaline reuptake inhibition enhances human exercise performance in warm, but not temperate conditions. *J. Physiol.* 565 873–883. 10.1113/jphysiol.2004.079202 15831540 PMC1464564

[B92] Wigfield A. Eccles J. S. (2000). Expectancy–value theory of achievement motivation. *Contemp. Educ. Psychol.* 25 68–81. 10.1006/ceps.1999.1015 10620382

[B93] Zając A. Chalimoniuk M. Gołaś A. Lngfort J. Maszczyk A. (2015). Central and peripheral fatigue during resistance exercise–a critical review. *J. Hum. Kinet.* 49 159–169. 10.1515/hukin-2015-0118 26839616 PMC4723165

